# PILA: Sub-Meter Localization Using CSI from Commodity Wi-Fi Devices

**DOI:** 10.3390/s16101664

**Published:** 2016-10-10

**Authors:** Zengshan Tian, Ze Li, Mu Zhou, Yue Jin, Zipeng Wu

**Affiliations:** Chongqing Key Lab of Mobile Communications Technology, Chongqing University of Posts and Telecommunications, Chongqing 400065, China; tianzs@cqupt.edu.cn (Z.T.); zhoumu@cqupt.edu.cn (M.Z.); jinyue063@gmail.com (Y.J.); wuzipeng2010@foxmail.com (Z.W.)

**Keywords:** indoor localization, Wi-Fi, Channel State Information, Angle-of-Arrival, Received Signal Strength

## Abstract

The aim of this paper is to present a new indoor localization approach by employing the Angle-of-arrival (AOA) and Received Signal Strength (RSS) measurements in Wi-Fi network. To achieve this goal, we first collect the Channel State Information (CSI) by using the commodity Wi-Fi devices with our designed three antennas to estimate the AOA of Wi-Fi signal. Second, we propose a direct path identification algorithm to obtain the direct signal path for the sake of reducing the interference of multipath effect on the AOA estimation. Third, we construct a new objective function to solve the localization problem by integrating the AOA and RSS information. Although the localization problem is non-convex, we use the Second-order Cone Programming (SOCP) relaxation approach to transform it into a convex problem. Finally, the effectiveness of our approach is verified based on the prototype implementation by using the commodity Wi-Fi devices. The experimental results show that our approach can achieve the median error 0.7 m in the actual indoor environment.

## 1. Introduction

With the increase of the demand for the ubiquitous Location-based Services (LBSs), localization and navigation applications become more important in daily life. In an outdoor environment, people prefer to use the Global Navigation Satellite System (GNSS) to achieve the LBSs, whereas the signal from the satellites cannot be easily received in indoor environment, which results in the failure of the GNSS. However, many LBSs significantly depend on the highly-accurate indoor localization such as when finding goods in the mall, locating in the mine, and rescuing.

Wi-Fi localization has great promise in the area of indoor localization due to the wide deployment of the commodity Wi-Fi devices. Up to now, existing Wi-Fi localization systems are mainly based on the Angle-of-arrival (AOA) or Received Signal Strength (RSS) measurement. The accuracy of the AOA measurement-based localization systems is around 0.4 m [1,2]. They measure the AOA of the signal from at least two Access Points (APs), and then use the triangulation algorithm to locate the target. By using the well-known Multiple Signal Classification (MUSIC) algorithm [3], each AP is equipped with six antennas for the AOA estimation since there are normally at most six significant signal paths in indoor environment [4]. Thus, the AOA measurement-based localization systems depend on the special hardware modification like the design of specific antennas, which is challenging and with significantly high cost. To solve this problem, we propose a new approach by using the Channel State Information (CSI) which is available in many existing commodity Wi-Fi devices to estimate the AOA of the multipath signal with small hardware modification.

At the same time, the RSS measurement-based localization systems have also become popular by using the existing commodity Wi-Fi devices. Although they are easy to be deployed, the corresponding localization error is about 3 m [5,6], which is much larger than the one by the AOA measurement-based localization systems. The RSS measurement-based localization systems generally involve two phases. In the first phase, the RSS measurements at each Reference Point (RP) are collected, and then used to construct the mapping relationship between the RSS measurements and the corresponding RPs, namely radio map. In the second phase, the Euclidean distance between the newly collected RSS measurements and pre-collected RSS measurements in radio map is calculated, and then the RPs with the RSS measurements corresponding to the smallest Euclidean distances are used to estimate the locations of the target. In this paper, we use both the AOA and RSS measurements to construct a new objective function for the indoor localization problem. On one hand, our approach improves the localization accuracy compare with the one by using the RSS or AOA measurement solely. On the other hand, it reduces the time and labor cost involved in the radio map construction significantly, and also exhibits well robustness to the environmental change.

**Figure 1:**
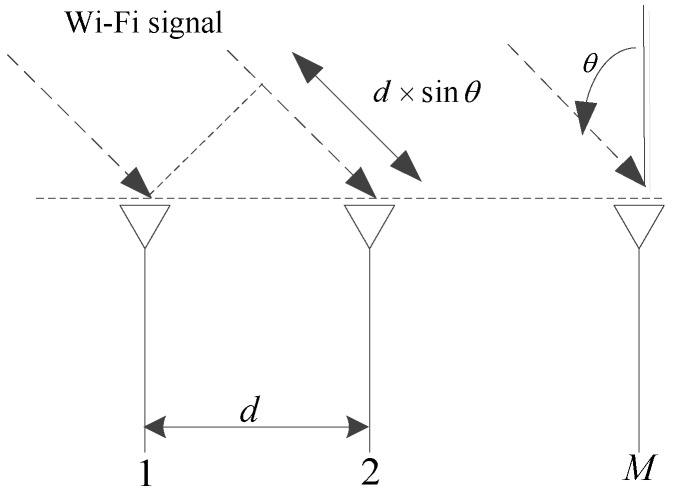
Wi-Fi signal arriving at linear antenna array.

In summary, the main contribution of this paper is to design a new precise indoor localization system based on the CSI from the commodity Wi-Fi APs to estimate the AOA of the multipath signal. To overcome the limitation of the conventional AOA estimation approaches, which generally require at least six antennas, we rely on the Orthogonal Frequency Division Multiplexing (OFDM) modulation property to estimate the AOA of the signal with only three antennas. Our approach is based on the fact that the multipath effect not only results in the measurable change of the CSI in the antenna array, but also changes the CSI across different sub-carriers due to the difference of the Time of Arrival (TOA) measurement. Specifically, when the signal with the AOA *θ* arrives at the linear antenna array which consists of *M* antennas, the difference of the propagation delay between every two neighboring antennas with the spacing distance *d* is d×sinθ, as shown in Figure 1. In addition, since the Wi-Fi signal is based on the OFDM modulation and the different subcarriers are with different frequencies, the different subcarriers will be featured with different accumulative phases with respect to the same TOA measurement.

In an indoor environment, the Wi-Fi signal from the same AP is generally correlated, which is not appropriate for the conventional MUSIC algorithm. To solve this problem, we propose a new two-dimensional spatial smoothing approach for the AOA estimation with respect to the multiple correlated signals. Furthermore, the CSI measured by the Wi-Fi Network Interface Card (NIC) suffers from the measurement error due to the imperfect signal processing by the hardware like the signal boundary detection, which introduces the additional time delay, namely Packet Detection Delay (PDD), to all the signal paths. Since different signal packets are with different PDD, the TOA measurement cannot capture the true time taken by the signal traveling from the APs to the target. In this paper, we use the CSI from multiple APs to estimate the AOA and TOA measurements of the multipath signal to locate the target. However, the most important problem is the identification of the direct signal path from the target to each AP correctly. The authors in [7] proposed to identify the direct signal path according to the TOA measurement, which may not be reliable in the multipath environment. The authors in [8] declared that the direct signal path is corresponding to the AOA measurement with the highest space spectrum value. However, in the indoor environment, the direct signal path may be weaker than the indirect path which resulting from the multi-path effect.

In this paper, based on the assumption that the signal on the reflected paths is generally with large variation of the AOA and TOA measurements comparing with the one on the direct path, we utilize the Gaussian means clustering algorithm to identify the direct signal path. Considering the fact that the error of the CSI results in the additional noise in the TOA estimation, we propose to use the phase sanitization algorithm to remove the interference of this error before estimating the AOA and TOA measurements.

Different from the existing indoor localization systems which integrate multiple types of measurements [9,10], we propose a new linear Least Square (LS)-based object function for the localization. In addition, since the localization problem is non-convex, we use the Second-order Cone Programming (SOCP) relaxation approach to convert it into a convex problem. Our findings are summarized as follows. First of all, the proposed system is easy to be deployed and with small hardware modification. Second, by using the commodity Wi-Fi APs with three antennas, the receiver is independent of the motion sensors like the gyroscope and accelerometer. Third, the proposed system achieves the median error 0.7 m and 68th error 1 m, which are smaller than the ones by the existing systems using the AOA or RSS measurement solely.

We briefly outline the organization of the rest of this paper. Section 2 gives some related work on the existing Wi-Fi localization systems. Section 3 describes the steps of the proposed system including the CSI-based AOA estimation, direct signal path identification, and target localization in detail. The performance of our system in an actual indoor environment is discussed in Section 4. Finally, we conclude the paper in Section 5.

## 2. Related Work

The existing Wi-Fi localization systems are mainly based on the propagation modeling [11] and location fingerprinting [12]. The systems using the propagation modeling locate the target based on the triangulation approach, while the ones using the location fingerprinting locate the target by constructing the mapping relationship between the RSS patterns and physical locations. One of the most representative location fingerprinting-based localization systems is the RADAR [13] which achieves the meter-level accuracy. However, it suffers from the time consuming and labor intensive process of radio map construction. There are some evolved RSS measurement-based localization systems which are independent of the huge time and labor cost [14,15,16], and meanwhile they are also robust to the environmental change.

At the same time, there are many fusion localization systems by using the data from the Wi-Fi module and motion sensors like the gyroscope, accelerometer, and magnetometer [17]. The systems using the motion sensors generally apply the Pedestrian Dead Reckoning (PDR) algorithm to achieve the continuous-time localization [18,19], but they are suffered by the accumulative error as the time goes. However, the fusion localization systems are limited for the application since many mobile devices are not embedded with motion sensors.

Since many off-the-shelf Wi-Fi APs supports the Multiple Input Multiple Output (MIMO) technique by using multiple antennas, the AOA measurement-based localization systems have been significantly developed. The ArrayTrack [20] requires the Wi-Fi APs to be equipped with at least seven antennas to estimate the AOA measurement. The Ubicarse [1] is based on the Synthetic Aperture Radar (SAR) technique to achieve the sub-meter localization accuracy. However, it requires the target to be equipped with a rotation antenna which cannot be satisfied by the commodity Wi-Fi devices. Recently, there are also some systems use the commodity Wi-Fi devices to estimate AOA measurement [21].

## 3. System Description

**Figure 2:**
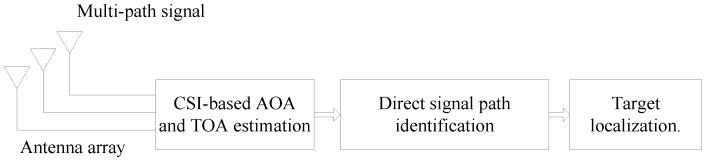
System description.

As shown in Figure 2, our system consists of three main steps as follows.

1. **CSI-based AOA estimation.** We estimate the AOA and TOA based on the CSI obtained from the existing commodity Wi-Fi APs with three antennas, and meanwhile employ the 2-D spatial smoothing algorithm to eliminate the interference of the coherent multi-path signal.
2. **Direct signal path identification.** We identify the direct signal path based on the likelihood of each cluster obtained by the Gaussian means clustering algorithm, and meanwhile use the phase sanitization algorithm to avoid the impact of the error of CSI measurement on the TOA estimation.
3. **Target localization.** We integrate the AOA and RSS measurements based on the LS criterion to construct a new object function for the localization problem.

**Figure 3:**
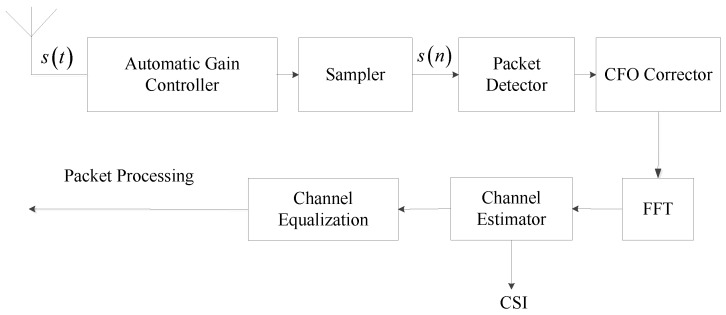
Flow chart of signal processing under the 802.11n NIC.

In our system, the transmitter is required to conform to the IEEE 802.11n standard for the sake of employing the spatial diversity technique with multiple antennas to achieve the high data transmission rate. Figure 3 shows the flow chart of signal processing under the 802.11n NIC. The incoming analog signal, $s\left(t\right)$, is processed by the Automatic Gain Controller (AGC) to compensate the signal amplitude attenuation, and then sampled as the discrete signal, $s\left(n\right)$. The packet detector and central frequency offset corrector (CFO) corrector are used to confirm the incoming packet and compensate the central frequency offset respectively. To extract the data correctly, the receiver applies the channel equalization to estimate the impact of the channel on each subcarrier [22]. The CSI obtained from the channel equalization involves the amplitude and phase information.

In an indoor environment, there are always many signal paths detected at the receiver due to various signal refraction and reflection. Different signal paths are featured with different attenuation and propagation delay, while the corresponding RSS measurements are generally assumed to obey the Gaussian distribution [23]. Based on the results in [24], we describe the Channel Frequency Response (CFR) as:

(1) $$h\left(f\right)=\sum_{l=0}^{L}\gamma_{l}\cdot e^{-j\cdot 2\pi \cdot f\cdot \tau_{l}}$$

where *L* is the number of signal paths. $\gamma_{l}$ and $\tau_{l}$ are the path coefficients and propagation delay with respect to the *l*th signal path respectively. The CSI is obtained by sampling the CFR with the sampling rate $F_{f}=1/\Delta f$ and Δf is the sampling interval. In our system, the selected Intel 5300 NIC reports the CSI of 30 subcarriers for each antenna.

### 3.1. Two-Dimensional Spatial Smoothing

By assuming that there are *K* signal paths including the direct and indirect ones, the CSI at three antennas can be described as:

(2) $$H\;={\left[h_{1,1},\cdots h_{1,30},h_{2,1},\cdots h_{2,30},h_{3,1},\cdots h_{3,30}\right]}^{T}$$

where H is a 90×1 vector and $h_{m,n}$ is the CSI of the *n*th subcarrier at the *m*th antenna. Based on Equations (1) and (2), we obtain

(3) H=AΓ+N

where $\Gamma ={\left[\gamma_{1}\cdots \gamma_{K}\right]}^{T}$ is a K×1 vector of the path coefficient with respect to the *K* signal paths and N is a 90×1 noise vector. A is the 90×K direction matrix and A equals to

(4) $$A=\left[a\left(\theta_{1},\tau_{1}\right),\cdots ,a\left(\theta_{k},\tau_{k}\right),\cdots ,a\left(\theta_{K},\tau_{K}\right)\right]$$

where $a\left(\theta_{k},\tau_{k}\right)$ is a 90×1 direction vector which is described as:

(5) $$a\left(\theta_{k},\tau_{k}\right)={\left[a_{1}\left(\theta_{k},\tau_{k}\right),a_{2}\left(\theta_{k},\tau_{k}\right),a_{3}\left(\theta_{k},\tau_{k}\right)\right]}^{T}$$

In Equation (5), $a_{m}\left(\theta_{k},\tau_{k}\right)$ is a 30×1 direction vector of the *k*th signal path at the *m*th antenna, as shown in Equation (6).

(6) $$a_{m}\left(\theta_{k},\tau_{k}\right)={\left[a_{1,m}\left(\theta_{k},\tau_{k}\right),\cdots ,a_{i,m}\left(\theta_{k},\tau_{k}\right),\cdots ,a_{30,m}\left(\theta_{k},\tau_{k}\right)\right]}^{T}$$

where $a_{i,m}\left(\theta_{k},\tau_{k}\right)=e^{-j\Delta \psi_{i,m}\left(k\right)}$ and $\Delta \psi_{i,m}\left(k\right)=2\pi \left(i-1\right)\Delta f\tau_{k}+2\pi d\left(m-1\right)\frac{sin\theta_{k}}{\lambda_{i}}$ is the phase difference of the *i*th$\left(i=1\cdots 30\right)$ subcarrier. Δf is the frequency interval of every two neighboring subcarriers. *d* is the physical distance between every two adjacent antennas. $\theta_{k}$ and $\tau_{k}$ are the AOA and TOA with respect to the *k*th signal path respectively.

**Figure 4:**
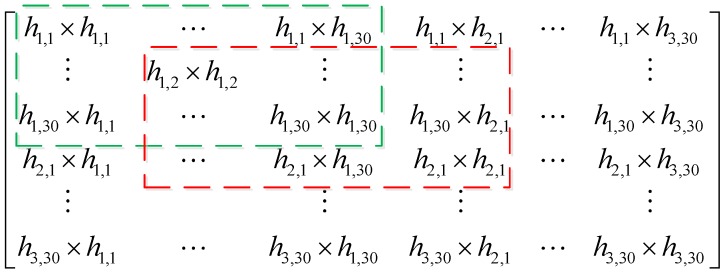
Process of 2D-SS on $R_{X}$.

The covariance matrix of the CSI in Equation (3) is calculated by:

(7) $$R_{x}=E\left\{HH^{H}\right\}$$

where $H^{H}$ is the conjugate transpose of H. Based on the result in [25], the eigenvectors corresponding to the noise are orthogonal to the direction vectors in A and the space spectrum of the AOA and TOA, *θ* and *τ*, can be described as:

(8) $$P_{music}\left(\theta ,\tau \right)=\frac{1}{a^{H}\left(\theta ,\tau \right)E_{N}E_{N}^{H}a^{H}\left(\theta ,\tau \right)}$$

where $E_{N}$ is the set of eigenvectors with respect to the noise subspace of $R_{X}$ To eliminate the interference of the coherent signals, we conduct the Two-dimensional Spatial Smoothing (2D-SS) [26], on $R_{X}$ instead of H, as shown in Figure 4. The elements in the dashed green and red boxes construct the covariance matrices of the first and second sub-arrays respectively. Based on the observation that the first elements of the covariance matrices of the first and second sub-arrays are $h_{1,1}\times h_{1,1}$ and $h_{1,2}\times h_{1,2}$, we get the covariance matrices of the existing sub-arrays by increasing the subcarrier ID and antenna index number to $L_{2}=30-N_{sub2}+1$, $L_{1}=3-N_{sub1}+1$ respectively, $N_{sub1}=2$ and $N_{sub2}=15$ are chosen. Then, the number of sub-arrays and elements in each sub-array equals to $L=L_{1}\times L_{2}$ and $N_{sub1}\times N_{sub2}$ respectively.

The covariance matrix of the CSI after the process of 2D-SS on $R_{X}$, $R_{2D-SS}$, is modified into

(9) $$R_{2D-SS}=\frac{1}{L_{1}L_{2}}\sum_{m=1}^{L_{1}}\sum_{n=1}^{L_{2}}R_{m,n}$$

where $R_{m,n}$ is the sub-covariance matrix in $R_{X}$ with respect to the *n*th subcarrier at the *m*th antenna. Then, we conduct the MUSIC algorithm on the smoothed covariance matrix of the CSI to obtain the direction vectors, as well as the AOA and TOA with respect to each signal path.

To verify the effectiveness of the 2D-SS in AOA estimation, two simulations are conducted as follows. We assume that there are 30 subcarriers operating at the 5.2 GHz with the spacing distance 1.25 MHz. The antenna array contains three antennas and the spacing distance between every two adjacent antennas, *d*, equals to λ/2, where *λ* is the wavelength of the signal. The impact of value *d* on the AOA estimation will be further discussed in the following section.

**Figure 5:**
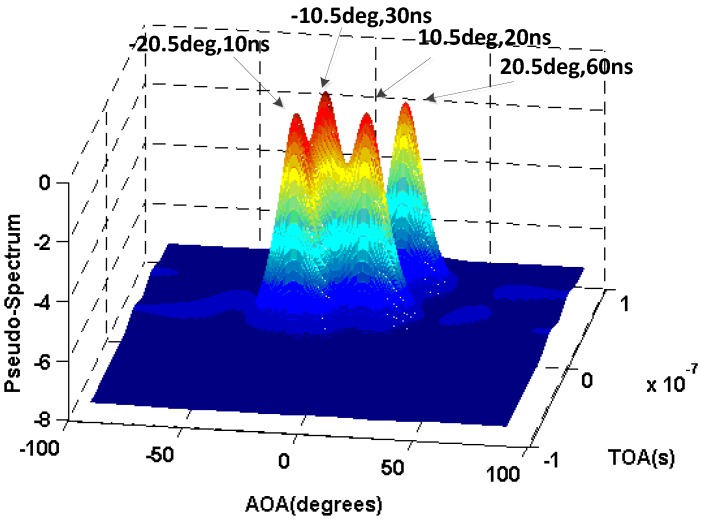
Result of AOA and TOA estimation for the incoherent signals.

In the first simulation, we assume that there are four incoherent signals with the AOA $-20^{\circ }$, $-10^{\circ }$, $10^{\circ }$, $20^{\circ }$ and TOA 10 ns, 30 ns, 20 ns, and 60 ns respectively. Figure 5 shows the results of the AOA and TOA estimation, from which we can find that by the 2D-SS, the estimated AOA and TOA are much similar to the real ones.

**Figure 6:**
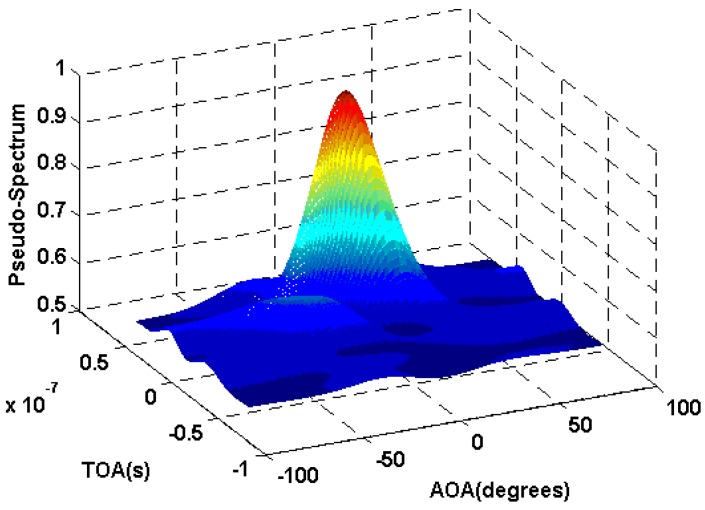
Result of AOA and TOA estimation for the coherent signals without the 2D-SS.

**Figure 7:**
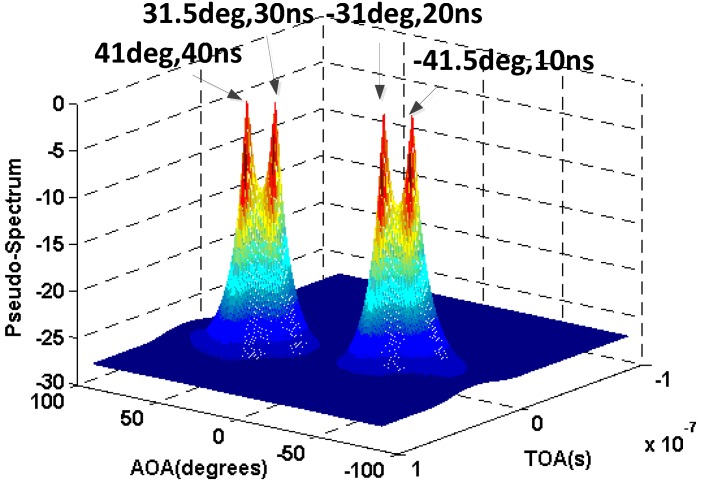
Result of AOA and TOA estimation for the coherent signals with the 2D-SS.

In the second simulation, we assume that there are four coherent signals with the AOA $-40^{\circ }$, $-35^{\circ }$, $35^{\circ }$, $40^{\circ }$ and TOA 10 ns, 20 ns, 30 ns, and 40 ns. Figure 6 and Figure 7 show the results of the AOA and TOA estimation without and with the 2D-SS respectively.

From these figures, we can conclude that the 2D-SS can not only effectively preserve but also significantly improve the accuracy of the AOA and TOA estimation for the incoherent and coherent signals respectively.

### 3.2. Direct Signal Path Identification

#### 3.2.1. Packet Detection Delay

To identify the direct signal path, some prior work [27,28] relies on the TOA measurement to claim that the signal path with the shortest TOA is most likely to be the direct one. However, the TOA estimation by using the raw CSI obtained from the Wi-Fi NIC cannot be accurate due to the channel distortion and hardware imperfection.

In a Wi-Fi network, to detect the packets, the receiver is required to sample the incoming signal. The process of signal sampling involves the Packet Detection Delay (PDD) since the starting boundary of the packets is unknown. By setting $n_{\xi }$ as the PDD, the impact of the PDD on the phase measurement is discussed as follows. Based on Equation (1), we conduct the IFFT (Inverse Fast Fourier Transform) to transform the CFR to the Channel Impulse Response (CIR), $f\left(t\right)$

(10) $$f\left(t\right)=\sum_{l=0}^{L}\gamma_{l}\delta \left(t-\tau_{l}\right)$$

where $\delta \left(\cdot \right)$ is the Delta function. Then, we conduct the discrete Fourier transform to obtain the discrete value of the CFR, $h\left(k\right)$

(11) $$h\left(k\right)=\sum_{n=0}^{N-1}f\left(n\right)e^{-j2\pi kn/N}$$

where $f\left(n\right)$ is the discrete value of the CIR. *k* is the frequency index. *N* is the length of the IFFT. By considering the PDD in $f\left(n\right)$, we convert Equation (11) into

(12) $$h\left(k\right)e^{-j2\pi kn_{\xi }/N}=\sum_{n=0}^{N-1}f\left[\left(n-n_{\xi }\right)\right]e^{-j2\pi kn/N}$$

In Equation (12), we can find that PDD adds a constant offset to the TOA estimates of all the paths, and this common additional delay manifests itself as a linear in frequency term in the phase response of the channel [29]. Hence, PDD results in adding $2\pi kn_{\xi }/N$ to the phase of the CFR value of *k*th sub-carrier.

#### 3.2.2. Phase Sanitization

In some prior work [20,30], it is found that the AOA of the direct signal path has smaller variation compare with the indirect ones. Based on this, we classify the peak points obtained from the MUSIC algorithm into different clusters and the clusters with the smaller variance of the AOA and TOA are selected to the candidates of the direct signal path. Since the PDD varies for different packets, the variation of the TOA is difficult to be obtained. To solve this problem, we propose the phase sanitization algorithm to avoid the impact of the PDD on different packets in the results that follow.

By setting $\phi_{m,n}$ as the phase of the CSI of the *n*th sub-carrier at the *m*th antenna, we aim to optimize the value ${\hat{\tau }}_{p}$ to construct a linear fitting model for the phase of the CSI to avoid the impact of the PDD on different packets. The linear fitting function is defined as:

(13) $$\varphi \left(n\right)=2\pi \Delta f\left(n-1\right)\mu +\beta$$

where *n* is the sub-carrier index. *μ* is the slope of the function. *β* is a constant. To optimize the linear fitting, we construct

(14) $${\hat{\tau }}_{p}=\underset{\mu }{\arg\min}\sum_{m}^{3}\sum_{n}^{30}{\left(\phi_{m,n}-\varphi \left(n\right)\right)}^{2}$$

To solve this optimal problem in Equation (14), we employ the well-known least square algorithm to get the optimal value ${\hat{\tau }}_{p}$ based on the CSI phase across 30 subcarriers at three antennas.

Then the phase of the CSI be modified into

(15) $${\hat{\phi }}_{m,n}=\phi_{m,n}+2\pi \Delta f\left(n-1\right){\hat{\tau }}_{p}$$

**Figure 8:**
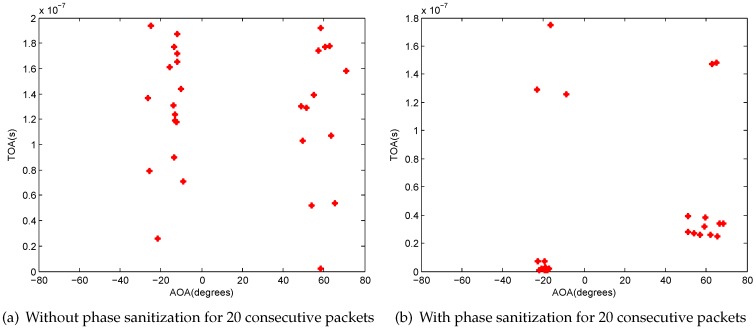
Results of AOA and TOA estimation.

Figure 8 shows a simple example about the AOA and TOA estimation with and without phase sanitization. The experimental setting is described in Section 4 in detail. Figure 8a,b show the results of AOA and TOA estimation without and with phase sanitization for 20 consecutive packets. From these figures, we can find that the phase sanitization algorithm not only preserves the result of AOA estimation, but also avoids the impact of the PDD on different packets. Our phase sanitization is similar to the data sanitization process in [29] and is an extension of the process to three antennas.

#### 3.2.3. Gaussian Means Clustering

After the peak points are obtained from the MUSIC algorithm, we use the Gaussian means clustering algorithm to identify the direct signal path.

Specifically, first of all, we randomly select *k* peak points as the *k* initial clustering centers, and then conduct the K-means clustering to obtain clusters. Second, we examine the distribution of the peak points in each cluster. If the peak points in a cluster do not approximately obey the Gaussian distribution, we randomly select two peak points in this cluster as the two new clustering centers, and then conduct the K-means clustering of the peak points in this cluster to obtain two new clusters. We continue this process until the peak points in every cluster approximately obey the Gaussian distribution or cannot be split further.

#### 3.2.4. Likelihood Assignment

From Section 3.2.2, we obtain that since the TOA of all the paths have the same delay due to the PDD, we use the phase sanitization method to remove the variance of the TOA, and meanwhile rely on the TOA information to identify the direct path which travels the shortest relative distance. To guarantee the robustness of our system, we select several paths with the relative short TOA as the candidates.

Since the number of peak points in the clusters which are corresponding to the real signal paths is larger than the one corresponding to the spurious paths, we rely on the probabilistic analysis to assign a likelihood value to each path. The likelihood value of each path is calculated by incorporating the number of peak points with the variance of the AOA and TOA.

(16) $$p_{k}=\omega_{n}n_{k}-\omega_{\theta }\sigma_{\theta_{k}}-\omega_{\tau }\sigma_{\tau_{k}}$$

where $n_{k}$, $\sigma_{\theta_{k}}$, and $\sigma_{\tau_{k}}$ are the number of peak points and the variance of the AOA and TOA in the cluster corresponding to the *k*th signal path. $\omega_{n}$, $\omega_{\theta }$, and $\omega_{\tau }$ are the weights of the number of peak points and the variance of the AOA and TOA.

**Figure 9:**
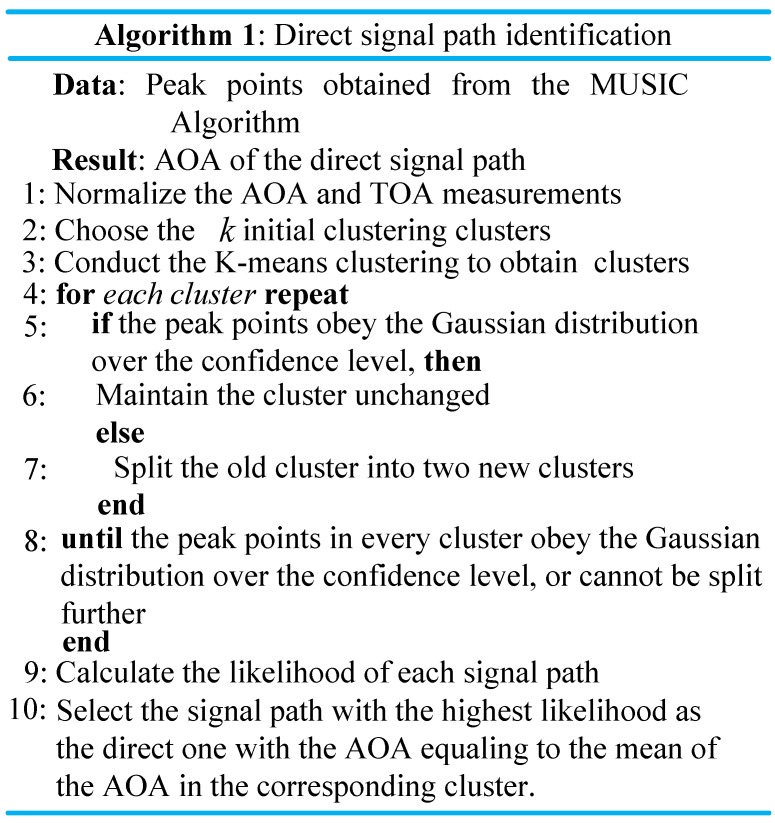
Direct signal path identification algorithm.

By using the concept of the likelihood of each signal path, the process of direct signal path identification is described in Figure 9.

### 3.3. Target Localization

To locate the target, we use the Second-order Cone Programming (SOCP) relaxation approach to transform the localization problem into a convex one based on the interior-point algorithm [31]. Specifically, by setting ${\mathrm{ff}}_{1},{\mathrm{ff}}_{2},\ldots ,{\mathrm{ff}}_{N}$ and p$\left(p,{\mathrm{ff}}_{i}\in R^{2},i=1,\cdots ,N\right)$ as the locations of the APs and target location, we estimate the distance between the target and each AP by

(17) $$d_{i}=10^{\frac{L_{i}}{10\gamma }},i=1,\cdots ,N$$

where $L_{i}=10\log_{10}\frac{P_{T}}{P_{i}}$(in dB). *γ* is the path loss exponent. $P_{T}$ and $P_{i}$ are the transmit power of the target and receiving power at the *i*th AP respectively.

**Figure 10:**
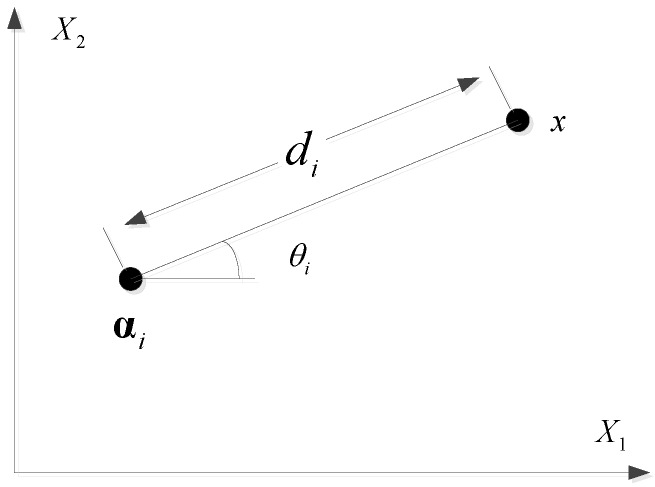
Geometrical relations between the target and the *i*th AP.

Based on the geometrical relations in Figure 10, the angle between the target and the *i*th AP is calculated by

(18) $$\theta_{i}=\arctan\left(\frac{x_{2}-\alpha_{i2}}{x_{1}-\alpha_{i1}}\right)$$

where $x=\left[x_{1},x_{2}\right]$ and ${\mathrm{ff}}_{i}=\left[\alpha_{i1},\alpha_{i2}\right]$ are the physical coordinates of the target and the *i*th AP. For simplicity, we assume that the APs and target are located with the same height. The impact of the height difference between the APs and target on the AOA estimation will be discussed further in the following section.

Based on the Least Square (LS) criterion, the estimated location of the target, $\hat{x}$, can be obtained by minimizing the objective function below.

(19) $$\hat{x}=\underset{x}{\arg\min}\sum_{i=1}^{N}{\left(∥p-{\mathrm{ff}}_{i}∥-d_{i}\right)}^{2}+\sum_{i=1}^{N}{\left(c_{i}^{T}\left(p-{\mathrm{ff}}_{i}\right)\right)}^{2}$$

where $c_{i}=\left[-\tan\left(\theta_{i}\right),1\right]$.

Problem in Equation (19) is obviously non-convex due to the second derivative of Equation (19) greater than zero and has no closed-form solution. To convert the problem in Equation (19) into a convex one, we first set the auxiliary variables $r_{i}=∥x-{\mathrm{ff}}_{i}∥$, $r=\left[r_{i}\right]$, $z=\left[z_{i}\right]$, and $g=\left[g_{i}\right]$, where $z_{i}=∥x-{\mathrm{ff}}_{i}∥-d_{i}$ and $g_{i}=c_{i}^{T}\left(x-{\mathrm{ff}}_{i}\right)$. Then, we obtain

(20) $$\begin{array}{c} \underset{x,z,g,h}{\mathrm{minimize}}{∥z∥}^{2}+{∥g∥}^{2}\\ s.t.\;\;r_{i}=∥x-{\mathrm{ff}}_{i}∥\;\;i=1,\cdots ,N\\ \;\;\;\;\;\;r_{i}=∥x-{\mathrm{ff}}_{i}∥\;\;i=1,\cdots ,N\\ \;\;\;\;\;\;z_{i}=r_{i}-d_{i},\;\;\;i=1,\cdots ,N\\ \;\;\;\;\;\;g_{i}=c_{i}^{T}\left(x-{\mathrm{ff}}_{i}\right),i=1,\cdots ,N \end{array}$$

In optimization, we can always assume that the objective is a linear function of the variables. This can be done via the epigraph representation of the problem, which is based on adding a new scalar variable. Based on [32] we set the epigraph variables $t_{1}$, $t_{2}$, and $t_{3}$, and we obtain

(21) $$\begin{array}{c} \underset{x,r,z,g,t_{1},t_{2}}{\mathrm{minimize}}t_{1}+t_{2}\\ s.t.\;r_{i}\leq ∥x-{\mathrm{ff}}_{i}∥\;\;i=1,\cdots ,N\\ \;\;\;\;\;z_{i}=r_{i}-d_{i},\;\;i=1,\cdots ,N\\ \;\;\;\;g_{i}=c_{i}^{T}\left(x-{\mathrm{ff}}_{i}\right),i=1,\cdots ,N\\ \;\;\;\;∥\left[\begin{array}{c} 2z\\ t_{1}-1 \end{array}\right]∥\leq t_{1}+1,∥\left[\begin{array}{c} 2g\\ t_{2}-1 \end{array}\right]∥\leq t_{2}+1 \end{array}$$

Based on Equation (21), we can find that the localization problem is transformed into a convex one which can be solved by using the CVX package [33].

## 4. Experimental Evaluation

The three main results obtained from the experimental evaluation are summarized as follows.

1. The proposed system achieves the median localization error 0.7 m, which is lower than the one by the systems using the RSS or AOA measurement solely.
2. The proposed AOA estimation approach achieves the median angle error within 5${}^{\circ }$, which is lower than the one by the conventional MUSIC algorithm.
3. The proposed direct signal path identification approach is with high identification precision, which enables to effectively reduce the large error probability.

### 4.1. Experimental Setup

**Figure 11:**
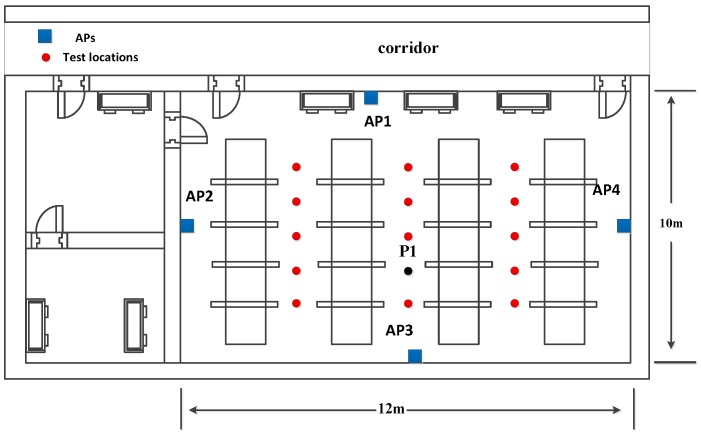
Geometrical structure of the testbed.

**Figure 12:**
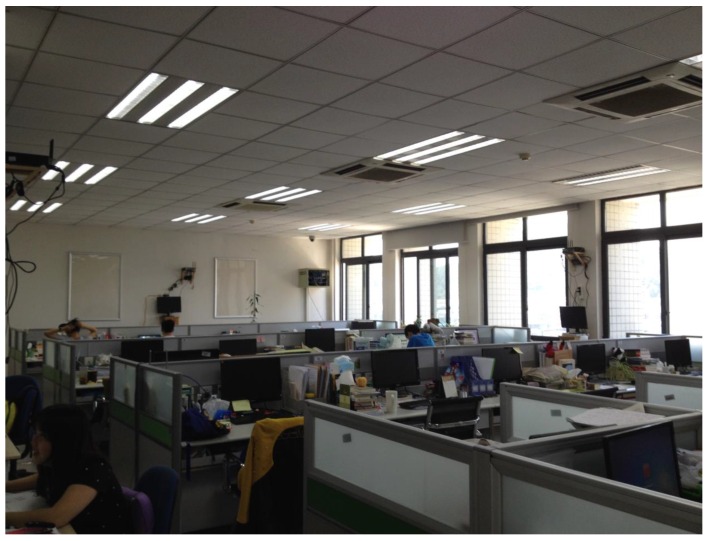
Picture of the testbed.

Figure 11 shows the geometrical structure of the testbed which is a typical lab environment and Figure 12 shows the picture of this lab. There are four APs and each of them is equipped with an Intel 5300 Wi-Fi NIC. The locations of APs are measured accurately by using a laser range finder. Another AP which is configured in the transmission mode is selected as the target to transmit the signal. We apply the Linux CSI toolkit [34] to collect the CSI at each AP, and then transmit it to the location server. All the calculations are executed in the location server by using the MATLAB.

### 4.2. Performance of AOA and TOA Estimation

**Figure 13:**
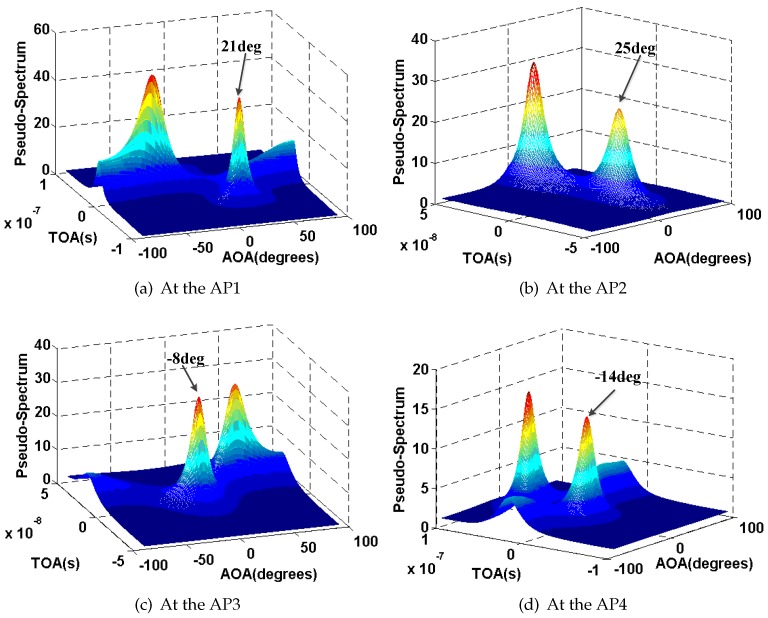
Results of AOA and TOA estimation.

We select the test location P1 (with a black circle in Figure 11) as an example to show the performance of the proposed AOA estimation approach in Figure 13. For the comparison, the AOA at the AP1, AP2, AP3, and AP4 is $19^{\circ }$, $21^{\circ }$, $-6^{\circ }$, and $-19^{\circ }$ respectively.

**Figure 14:**
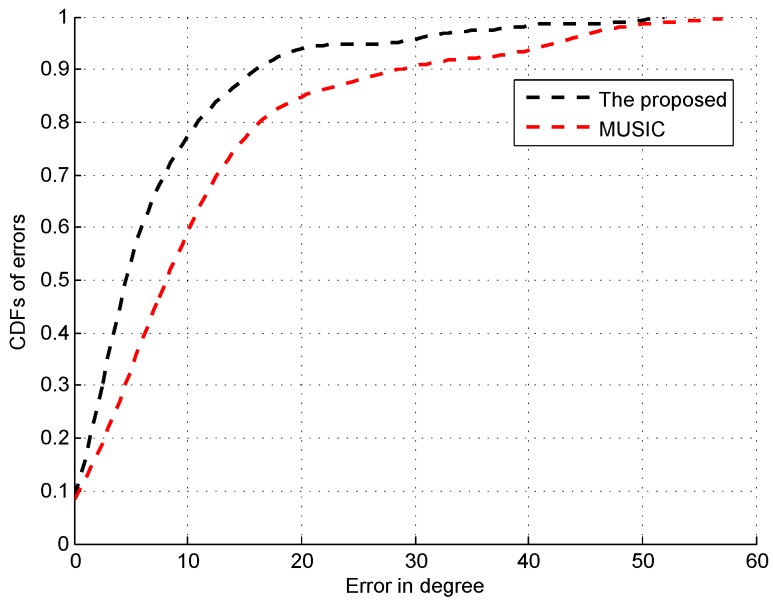
CDFs of errors of AOA estimation.

Figure 14 shows the CDFs of errors of AOA estimation by using the proposed and conventional MUSIC algorithms without spatial smoothing. The median and 80% errors by the proposed algorithm are about $4^{\circ }$ and $11^{\circ }$, which are much smaller than the ones by the MUSIC algorithm.

### 4.3. Performance of Direct Signal Path Identification

**Figure 15:**
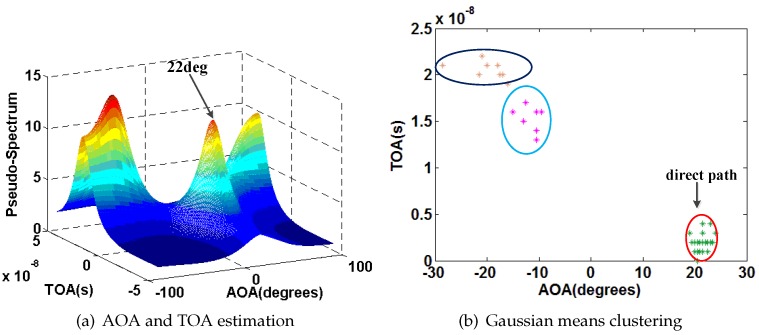
Results of AOA and TOA estimation and the related Gaussian means clustering at the AP1.

**Figure 16:**
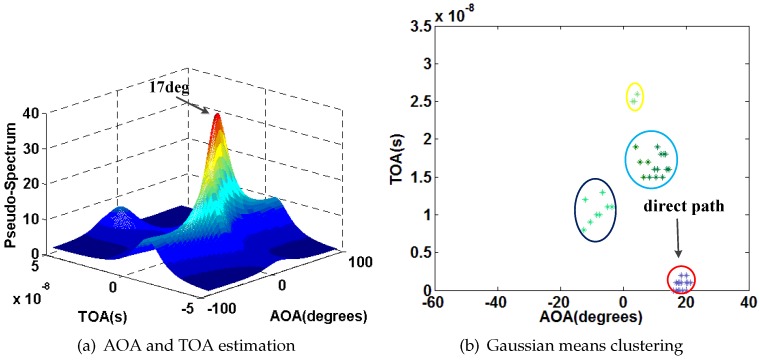
Results of AOA and TOA estimation and the related Gaussian means clustering at the AP2.

**Figure 17:**
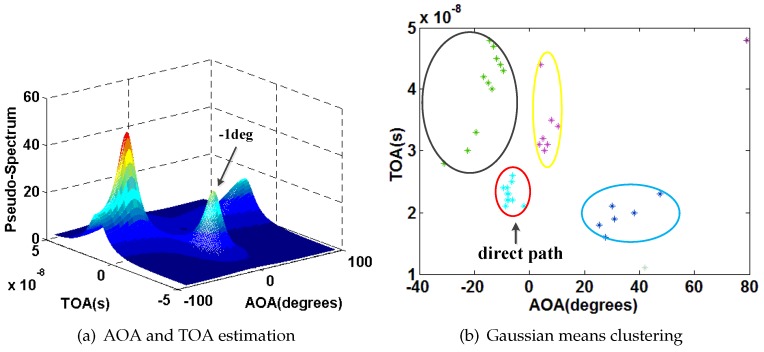
Results of AOA and TOA estimation and the related Gaussian means clustering at the AP3.

**Figure 18:**
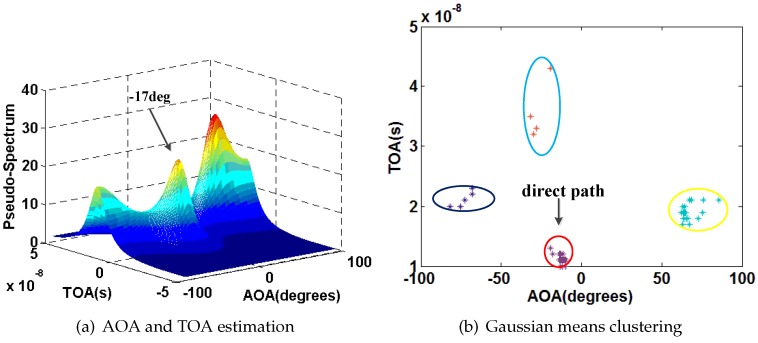
Results of AOA and TOA estimation and the related Gaussian means clustering at the AP4.

We also select P1 as an example. Figure 15, Figure 16, Figure 17 and Figure 18 show the results of AOA and TOA estimation and the related Gaussian means clustering with phase sanitization at each AP.

**Figure 19:**
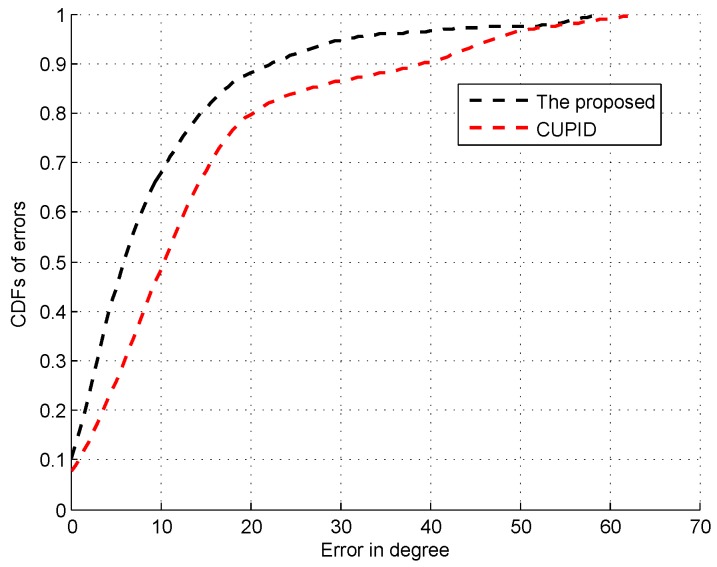
CDFs of errors of AOA estimation.

From these figures, we can find that the peak with the highest spectrum value is not always corresponding to the direct signal path since the direct signal path in the indoor environment is sometimes with the weaker RSS compared with the indirect ones due to the multi-path effect. Thus, the systems like the CUPID [8] select the AOA with the highest spectrum value as the one of the direct signal path is not accurate. To illustrate this result clearer, we compare the performance of AOA estimation by the proposed approach with the CUPID in Figure 19. From this figure, we can find that the proposed approach achieves the probability of the errors within $10^{\circ }$ 68%, which is higher than the one by the CUPID.

### 4.4. Impact of Height Difference on AOA Estimation

**Figure 20:**
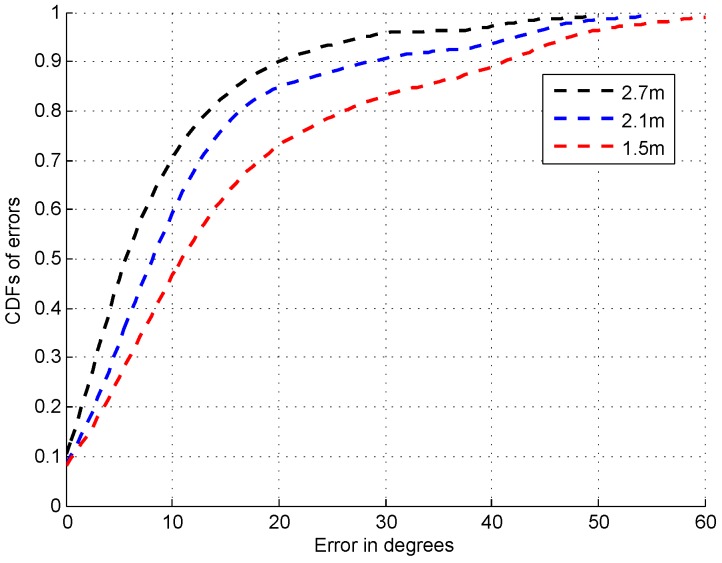
CDFs of errors of AOA estimation under different height of the target.

Since the APs and target are generally not placed with the same height in indoor environment, the conventional MUSIC algorithm requires the special shape of antenna array like the L-shape [35] for the AOA estimation. However, our system can overcome the problem of the requirement of the special shape of antenna array by using multiple APs for the localization. Figure 20 shows the CDFs of errors of AOA estimation by placing the APs with 2.7 m height and target with 2.7 m, 2.1 m, and 1.5 m height respectively. From this figure, we can find that when the APs and target are placed with the same height, the median and 70% errors are within $5^{\circ }$ and $10^{\circ }$. With the increase of the height difference between the APs and target, the error of AOA estimation increases as expected. For example, the median error and probability of the errors within $10^{\circ }$ increases from $8^{\circ }$ to $10.5^{\circ }$ and decreases from 60% to 47% as the height difference increases from 0.6 m to 1.5 m.

By placing the AP and target with 2.7 m and 1.5 m height respectively, we continue to investigate the CDFs of errors of AOA estimation as the distance between the AP and target, *d*, increases from 3 m to 9 m with the interval 3 m. We place the target at the location with different AOA with respect to the AP for each testing. Table 1 shows the median error of AOA estimation. From Table 1, we can find that as the value *d* increases, the median error of AOA estimation decreases. Comparing to the result of AOA estimation with no height difference, the additional error of AOA estimation caused by different value *d* is less than 3${}^{\circ }$.

### 4.5. Location Estimation

In our system, the target sends 200 signal packets in each second to the APs operating in the monitor mode, and then the APs transmit the CSI to the location server. After that, we use the previous 30 packets to form a dataset for the testing. To examine the localization performance of our system, we compare it with the existing ones by using the AOA [2] or RSS [13] measurement solely.

**Figure 21:**
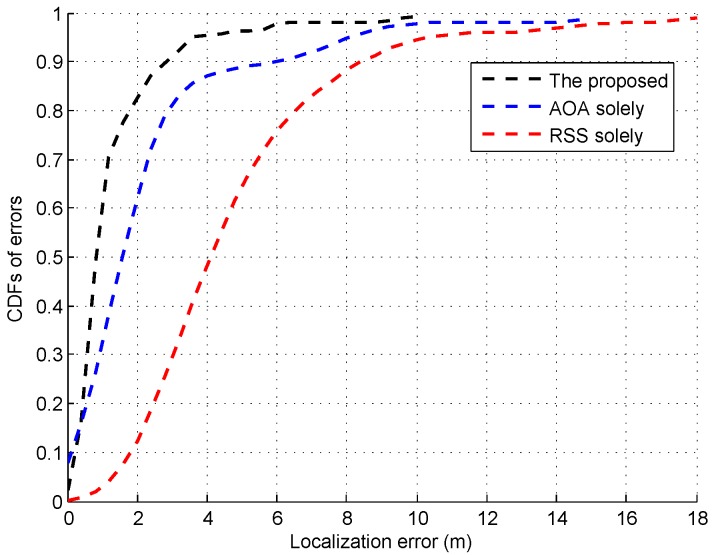
CDFs of errors by using the proposed system and the ones using the AOA or RSS solely.

As shown in Figure 21, the median error by the proposed system is 0.7 m, which is smaller than the one by the systems using the AOA or RSS measurement solely. Thus, our system is verified to be able to achieve the sub-meter localization accuracy without any special hardware modification.

### 4.6. Impact of AP Number

**Figure 22:**
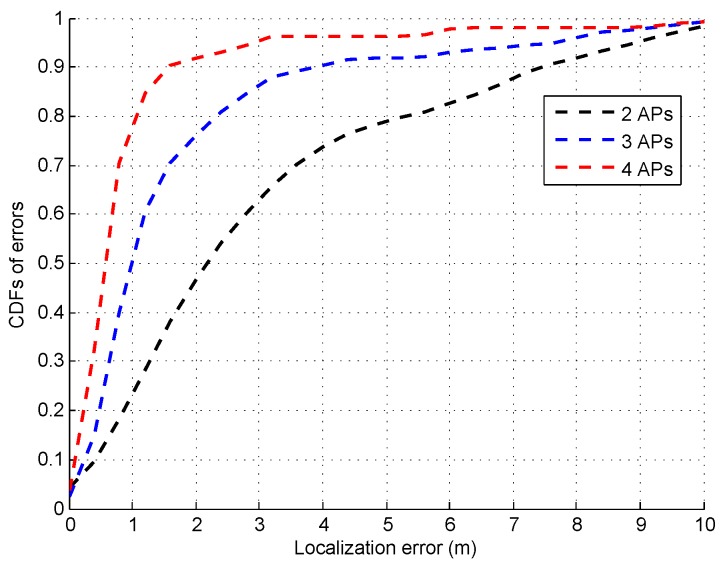
CDFs of errors under different AP number.

To investigate the impact of AP number on the localization accuracy of the proposed system, Figure 22 shows the CDFs of errors as the AP number increases from 2 to 4. As expected, the increase of AP number improves the localization accuracy. For example, the median error is 2 m, 1 m, and 0.7 m under the two, three, and four APs conditions, respectively.

### 4.7. Impact of Packet Number

**Figure 23:**
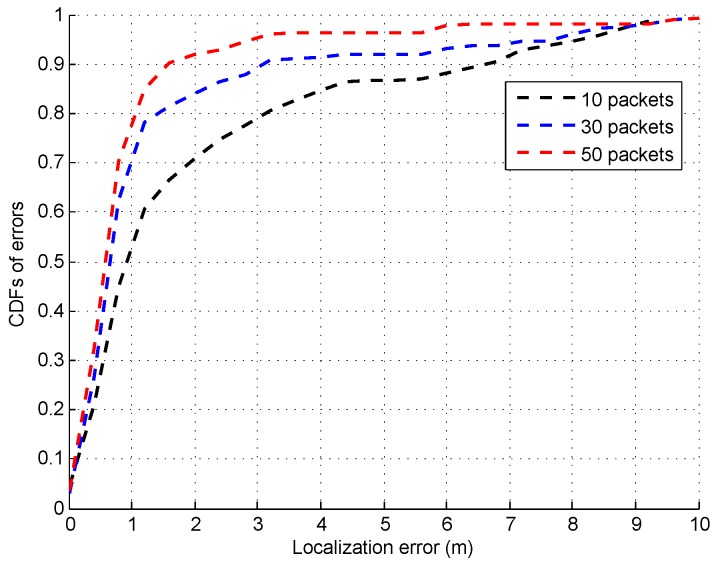
CDFs of errors under different packet number.

Since our system uses multiple signal packets to identify the LOS and NLOS signal paths, the packet number probably affects the precision of the direct signal path identification. On account of this, Figure 23 shows the CDFs of errors as the packet number increases from 10 to 50. From this figure, we can find that the localization accuracy changes slightly with the variation of packet number. For example, when the packet number increases from 10 to 50, the median error decreases from 0.8 m to 0.64 m respectively.

### 4.8. System Latency

**Figure 24:**
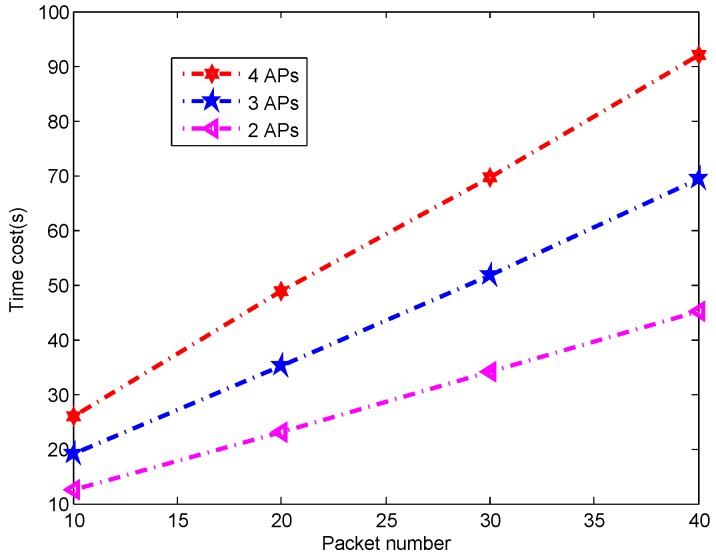
Time cost under different packet and AP number.

To evaluate the System latency on the localization, we varied the number of packets and APs and record the time cost. From Figure 24 we can find that the packet number used for the localization increases from 10 to 40 the time cost increases from 12.5 s to 45.2 s respectively when we use 2 APs to locate the target. The more APs we used to locate the target the more time we needed when the data process is serial. As expected, the increase of AP number takes more time to locate the target. For example, when we use 20 packets the time cost is 23.1 s, 35.2 s, and 48.7 s under the two, three, and four APs conditions, respectively.

## 5. Conclusions

The proposed indoor localization system uses the commodity Wi-Fi devices with three antennas to precisely estimate the AOA between the APs and target, and eventually achieve the sub-meter localization accuracy without any special hardware modification. By integrating the AOA and RSS measurements to locate the target, the localization performance of our system is significantly improved compared with the conventional ones by using the AOA or RSS measurement solely. Our system leverages the benefit of OFDM modulation property, and thereby it can be easily applied to the future 5G communication system.

## Figures and Tables

**Table 1:** The median AOA estimation errors with different *d*.

AOA	*d* = 3 m	*d* = 6 m	*d* = 9 m
–20${}^{\circ }$	$8^{\circ }$	$6^{\circ }$	$5^{\circ }$
20${}^{\circ }$	$7^{\circ }$	$6^{\circ }$	$4^{\circ }$
